# Relative deprivation and risk factors for obesity in Canadian adolescents

**DOI:** 10.1016/j.socscimed.2016.01.039

**Published:** 2016-01-28

**Authors:** Frank J. Elgar, Annie Xie, Timo-Kolja Pförtner, James White, Kate E. Pickett

**Affiliations:** a Institute for Health and Social Policy, McGill University, Montreal, Canada; b Institute for Medical Sociology, University of Cologne, Cologne, Germany; c School of Medicine, Cardiff University, Cardiff, Wales, UK; d Department of Health Sciences, University of York, York, England, UK

**Keywords:** Adolescence, Relative deprivation, Yitzhaki index, Physical activity, Food choices, Health behaviour in school-aged children

## Abstract

Research on socioeconomic differences in overweight and obesity and on the ecological association between income inequality and obesity prevalence suggests that relative deprivation may contribute to lifestyle risk factors for obesity independently of absolute affluence. We tested this hypothesis using data on 25,980 adolescents (11–15 years) in the 2010 Canadian Health Behaviour in School-aged Children (HBSC) study. The Yitzhaki index of relative deprivation was applied to the HBSC Family Affluence Scale, an index of common material assets, with more affluent schoolmates representing the comparative reference group. Regression analysis tested the associations between relative deprivation and four obesity risk factors (skipping breakfasts, physical activity, and healthful and unhealthful food choices) plus dietary restraint. Relative deprivation uniquely related to skipping breakfasts, less physical activity, fewer healthful food choices (e.g., fruits, vegetables, whole grain breads), and a lower likelihood of dieting to lose weight. Consistent with Runciman's (1966) theory of relative deprivation and with psychosocial interpretations of the health consequences of income inequality, the results indicate that having mostly better off schoolmates can contribute to poorer health behaviours independently of school-level affluence and subjective social status. We discuss the implications of these findings for understanding the social origins of obesity and targeting health interventions.

## Highlights

• We examined the social and behavioural determinants of obesity in adolescents.
• Data were collected in the WHO Health Behaviour in School-aged Children study.
• The Yitzhaki index of relative deprivation was calculated using material assets.
• Relative deprivation related to multiple lifestyle risk factors for obesity.
• They included skipping breakfasts, physical inactivity, and poor food choices.

## 1 Introduction

Rising trends in childhood obesity are a public health concern in many countries (Lobstein et al., 2004). The prevalence of overweight and obesity in young people in developed countries has increased by 47.1% between 1980 and 2013 (Ng et al., 2014), with a levelling off found recently in the United States (Ogden et al., 2014) and some countries in Europe and Asia (Olds et al., 2011). In 2011, approximately one-third of Canadian children and adolescents were estimated to be either overweight or obese (Roberts et al., 2012). Most youth do not outgrow this problem. About four out of five adolescents who are obese will continue to be obese as adults (Freedman et al., 2005). Obesity in children and youth increases the risks of type 2 diabetes, hypertension, sleep apnea, and cardiovascular disease (Biro and Wien, 2010, Freedman et al., 2007), and relates to a diminished quality of life (Swallen et al., 2005), low self-esteem (Strauss, 2000), social discrimination (Puhl and Brownell, 2001), and various psychiatric disorders (Mustillo et al., 2003).

Prior research on the social determinants of obesity has studied its complex relation to socioeconomic position (SEP). A socioeconomic gradient in overweight and obesity has been observed in many high-income countries whereby weight problems are more common in lower SEP groups (Devaux and Sassi, 2013, McLaren, 2007). The evidence from longitudinal studies suggests that this association is transactional; childhood obesity limits social mobility and prospectively relates to fewer years of education and lower incomes in adulthood (Gortmaker et al., 1993). Conversely, low SEP in childhood increases the risk for adult obesity even after differences in adult SEP are taken into account (Power et al., 2005, Senese et al., 2009).

The mechanisms that underlie this socioeconomic pattern involve material and psychosocial factors of affluence and social position (Adler and Ostrove, 1999). First, low SEP can impact health through limiting access to material resources that support health, such as affordable, nutritious foods and safe and accessible areas that facilitate physical activity (Conrad and Capewell, 2012), although some have argued that this material path stems mostly from individual SEP and not neighbourhood deprivation (Macintyre, 2007). Second, low SEP carries with it the psychosocial effects of low socioeconomic position in society and the stress and anxieties of living in relative poverty (Wilkinson and Pickett, 2007). This psychosocial path explains why the socioeconomic gradient in excess body mass extends throughout the full range of SEP including middle and upper SEP groups and why two individuals with equivalent material resources (e.g., household income) might differ in health status if one is surrounded by more affluent people and the other is surrounded by less affluent (Eibner and Evans, 2005, Elgar et al., 2013).

Research on the theoretical construct of relative deprivation has helped to unpack this psychosocial pathway by examining how upward socioeconomic comparisons can generate psychological stress and compromise health and wellbeing independently of absolute deprivation (Eibner and Evans, 2005). Relative deprivation, or “poverty amid plenty,” has been studied at the ecological level in terms of the correlation between income inequality and prevalence of adult obesity (Pickett et al., 2005, Wilkinson and Pickett, 2007) and between income inequality and adolescent body mass (Elgar et al., 2015). Although absolute deprivation could limit access to nutritional education and other health resources, the stress associated with feeling deprived in relation to others may explain why lower SEP groups also show less dietary restraint and dietary preferences for high fat and high caloric foods (Oliver and Wardle, 1999, Roemmich et al., 2002, Torres and Nowson, 2007).

The psychosocial dimension of SEP has also been investigated using subjective measures of perceived status or rank in the socioeconomic structure (Goodman et al., 2007). Health surveys of adolescents have found that subjective ratings of social position share weak but statistically significant associations with physical activity, healthful food choices, and reduced risk of obesity (Goodman et al., 2003, Quon and McGrath, 2014a). However, this research has also found that objective measures of SEP do not fully account for the association between subjective status and adolescent health, either because it is a distinct causal pathway to health or because its subjective nature allows it to share bidirectional effects on health (Garbarski, 2010). In Quon and McGrath's (2014b) meta-analysis of 44 studies on the effects of subjective social status on adolescent health, the effects of subjective status largely depended on the health domain with larger effects found on subjective health assessments than on specific health behaviours and physiological indicators of allostatic load. Using both objective and subjective measures of SEP might therefore give a deeper understanding of how relative differences in SEP – real or perceived – are associated with the behavioural determinants of obesity.

In a global context of a high prevalence of obesity (Ng et al., 2014), rising income inequality (Organisation for Economic Co-operation and Development, 2011), and widening socioeconomic differences in adolescents' body mass and physical activity (Elgar et al., 2015), the goal of this study was to better understand the contribution of relative deprivation to shaping social inequalities in obesity risk factors. To achieve this, we examined three socioeconomic variables - absolute deprivation between schools, relative deprivation within schools, and subjective social status - in relation to a set obesity risk factors in adolescents. We hypothesised that relative deprivation relates to physical inactivity, skipping breakfasts, fewer healthful food choices, more unhealthful food choices, and to less dietary restraint after other individual differences in body mass, absolute affluence, and subjective social status are controlled.

## 2 Method

### 2.1 Participants

The 2010 Health Behaviour in School-aged Children (HBSC) study in Canada surveyed 26,069 students in grades 6 to 10 in all provinces and territories except New Brunswick and Prince Edward Island (Freeman et al., 2011). Following an international protocol (Currie et al., 2012), a stratified sample of 436 schools was recruited to represent the distribution of regions, economic conditions, school types (public or Catholic), languages of instruction (English or French), and community sizes in Canada. Private schools, special needs schools and schools for youth in custody were excluded. The HBSC protocol stipulated a standard questionnaire format, item order, and testing conditions. Teachers or trained interviewers distributed the questionnaires in classroom settings.

The age of the sample ranged from 9 to 19 (mean = 13.85; SD = 1.52) years, and males (49.17%) and females (50.83%) were equally represented. Participation in the HBSC study was voluntary. School jurisdictions and individual schools chose to request either active or passive parent consent. Approximately 41% of participating schools used passive consent and 59% used active consent. Response rates were 11/13 (84.6%) at the provincial/territorial level, 436/765 (57.0%) at the school level and 26,078/33,868 (77.0%) at the individual level. Reasons for nonparticipation were failure to return consent forms, failure to receive parental consent, and absence on the day of survey administration. A university research ethics board approved the study procedures.

### 2.2 Measures and procedures

Teachers or trained interviewers administered the HBSC questionnaire in classroom settings. The survey collected data on sociodemographic characteristics and various health indicators and health-related behaviours. Of relevance to the present study are students' date of birth, gender, and self-reported body weight (*kg*) and height (*cm*). These variables were used to include the body mass index (*kg/m*^2^) as a control variable in the analyses. We did not attempt to identify overweight and obese cases given the high rate of misclassification that occurs with self-reported height and weight (Elgar et al., 2005).

Physical activity was measured with the question: “Over the past 7 days, on how many days were you physically active for a total of at least 60 min per day?” with responses ranging from 0 to 7 days. The question was prefaced with the description of “any activity that increases your heart rate and makes you get out of breath some of the time” followed by specific examples (e.g., running, brisk walking, skating, biking) to ensure the item measured moderate-to-vigorous physical activity. The criterion of 60 min per day was consistent with Canadian Physical Activity Guidelines for young people to be considered physically active (Tremblay et al., 2011). This measure of physical activity was found to have adequate test-retest reliability and concurrent validity alongside accelerometer data (Prochaska et al., 2001).

Two items measured the frequency of eating breakfasts, on weekdays and on weekends, and the responses were combined to determine the number of breakfasts per week (0–7). Skipping breakfasts based on similar self-report measures has well documented links to excess body weight in youth due to poorer nutrition and appetite control (Timlin et al., 2008).

A series of ten items on food choices asked “How often do you eat (a) fruit; (b) vegetables; (c) dark green vegetables; (d) orange vegetables; (e) whole grain breads; (f) sweets, candy or chocolate; (g) French fries (chips); (h) potato chips (crisps); (i) Coke or soft drinks with sugar; (j) cake or pastries (1 = never, 2 = less than once a week, 3 = once a week, 4 = 2–4 days a week, 5 = 5–6 days a week, 6 = once a day, 7 = more than once a day)?” These items were part of the HBSC Food Frequency Questionnaire and have been found to have good validity in relation to 24-h and 7-day food diary tools (Vereecken and Maes, 2003, Wong et al., 2012).

Dietary restraint was measured with the item: “At present, are you on a diet or doing something else to lose weight (1 = no, my weight is fine; 2 = no, but I should lose some weight; 3 = no, because I need to put on weight; 4 = yes, I am dieting to lose weight)?” The item has been used in the HBSC international survey since 2001/02, and early tests of its validity showed that it had good agreement with open-ended questions about dieting practices among youth in Finland and Belgium Flanders (Currie et al., 2001).

Subjective social status was measured using a survey item: “How well off do you think your family is (1 = Not at all well off, 2 = Not very well off, 3 = Average, 4 = Quite well off, 5 = Very well off)?” Estimates of absolute and relative deprivation were based on data collected with the HBSC Family Affluence Scale, a four-item index of common material assets, “Does your family own a car, van or truck (0 = no, 1 = yes, one, 2 = Yes, two or more)? During the past 12 months, how many times did you travel away on holiday (vacation) with your family (0 = not at all, 1 = once, 2 = twice, 3 = more than twice)? Do you have your own bedroom for yourself (0 = no, 1 = yes)? How many computers does your family own? (0 = none, 1 = one, 2 = two, 3 = more than two)?” Prior research found that the summation of scores on the Family Affluence Scale are a valid, reliable, and age-appropriate measure of family affluence in Canadian adolescents (Boudreau and Poulin, 2009, Currie et al., 2008) and less affected by nonresponse bias than longer socioeconomic assessments that request data on household income or parental occupation (Currie et al., 2008).

### 2.3 Derived variables

The data were analysed using Stata 14.1 (StataCorp LP, College Station, TX). Stata's *egen* function *zanthro* converted the body mass index (*kg/m*^*2*^) to standard deviation units (*z*BMI), which represented deviations from age- and gender-adjusted international norms according World Health Organisation child growth standards (Vidmar et al., 2013). The food choice items were combined to create composite indices of healthful food choices (fruit, vegetables, dark green vegetables, orange vegetables, and whole grain breads) and unhealthful food choices (sweets, candy or chocolate, French fries [chips], potato chips [crisps], Coke or soft drinks with sugar, and cake or pastries). We calculated the average response in each item set, resulting in scores that ranged from 1 (least frequent) to 7 (most frequent). As scales, these composite indices showed adequate internal consistency; α = 0.79 (healthful food choices) and α = 0.76 (unhealthful food choices).

The two deprivation variables were based on a reversed summary score of the HBSC Family Affluence Scale, which ranged from 0 (most deprived) to 9 (least deprived). Relative deprivation was estimated using the Yitzhaki index (Adjaye-Gbewonyo and Kawachi, 2012, Yitzhaki, 1979), which was modified by Subramanyam et al. (2009). Schoolmates represented the social reference group. The Yitzhaki index estimates the average difference in family affluence scores between the individual (*i*) and *N* schoolmates that had higher scores (*j*):

$$\text{Yitzhaki}\;{\text{Index}}_{i}=\frac{1}{N}\sum_{j}\left(y_{j}-y_{i}\right),\forall \left(y_{j}>y_{i}\right)$$

We could not include individual absolute deprivation in our regression analysis due to its close correlation with relative deprivation (*r* = 0.91). Therefore, differences in absolute deprivation were partially controlled by entering the average absolute deprivation score of each school to our models. The analysis excluded 98 students in 15 small schools that had fewer than 10 observations, given that schools were the social reference group used to estimate relative deprivation. With these cases excluded, the number of observations per school ranged from 10 to 330 students (mean = 81.23, SD = 61.56).

### 2.4 Data analysis

We used Stata's complex survey (SVY) commands to adjust standard errors according to the sampling design effect of school clusters, and multiple imputation (MI) commands to handle missing data on the four Family Affluence Scale items (7.23–7.97%), height (15.92%), and weight (15.12%). The probability of having missing data on these variables did not appear to relate to any other variable so we assumed the data were missing at random and did not specify auxiliary variables during the imputation (Rubin, 1987). Two hundred iterations of a Markov Chain Monte Carlo algorithm used age, gender, and the dependent variables to replace missing values with 25 plausible values (Schafer, 1997). School-level clustering could not be specified in the imputation due to the large number of clusters in the sample and variability in their sizes. Then, using SVY and MI modules together, we fitted linear regression models of physical activity, breakfasts, healthful food choices, and unhealthful food choices, and a multinomial (unordered logit) regression model of dietary restraint. Poststratification weights were applied to these analyses to ensure that the results accurately reflected the population of students in the Canadian regions represented in the study.

## 3 Results

Descriptive statistics on the main variables used in the study are shown in Table 1. The sample averaged 4.49 (SD = 2.03) days per week of 60+ minutes of physical activity and 5.41 (SD = 2.14) breakfasts per week. The distribution of adolescents' food choices varied between food types. As shown in Table 2, fruit and vegetable consumption was more common than unhealthy options, such as French fries (chips), potato chips (crisps), or soft drinks. Average healthful food choices was significantly higher (mean = 4.51, SD = 1.18) than unhealthful food choices (mean = 3.18, SD = 1.01), *t*(25,150) = 116.36, *p* < 0.001. With respect to weight loss behaviours, 12.63% (95% CI = 11.90, 13.41) of adolescents reported to be currently dieting to lose weight, 18.61% (95% CI = 17.74, 19.52) reported that they were not dieting but needed to lose weight, 7.82% (95% CI = 7.33, 8.33) reported that they were not dieting because they needed to put on weight, and the remaining 60.94% (95% CI = 59.64, 62.22) reported that they were not currently dieting to lose weight.

In testing the associations between relative deprivation and each obesity risk factor, we controlled the variation owing to school clustering, gender, age, *z*BMI, school-level absolute deprivation, and subjective social status. Table 3 shows linear regression analyses of physical activity, breakfasts, and healthful and unhealthful food choices. With all the variables entered into the models simultaneously, relative deprivation within schools uniquely related to less physical activity, fewer breakfasts, and fewer healthful food choices. However, relative deprivation did not relate to unhealthful food choices. Across the full range of relative deprivation, we observed differences of 0.84 fewer breakfasts per week, 1.20 fewer days of physical activity, and a 0.75-point difference on the 6-point scale of healthy food choices. Relative deprivation also related to less dietary restraint (Table 4) but the association was marginally significant (odds ratio = 0.94, 95% CI: 0.88, 0.99). Each 1-point increase in relative deprivation corresponded to a 6% reduction in the odds ratio of dieting (versus not dieting, “weight is fine”). Together, all the variables in these models explained approximately 4–15% of the variation in obesity risk factors.

Table 3, Table 4 also show the associations with school-level absolute deprivation and subjective social status. School-level absolute deprivation related to fewer days of physical activity, fewer breakfasts, and less healthful and more unhealthful food choices. School-level absolute deprivation did not relate to current dieting but positively related to not dieting despite wanting to lose weight (odds ratio = 1.19, 95% CI = 1.03, 1.39) and to not dieting and wanting to gain some weight (odds ratio = 1.22, 95% CI = 1.07, 1.40).

Subjective social status showed a similar pattern of associations but in the reverse. Higher subjective social status related to more physical activity and breakfasts, more frequent healthful food choices and less frequent unhealthful food choices. Subjective social status negatively related to dieting (odds ratio = 0.85, 95% CI: 0.80, 0.90), to not dieting but wanting to lose some weight (odds ratio = 0.83, 95% CI = 0.79, 0.88) and to not dieting and wanting to gain weight (odds ratio = 0.87, 95% CI = 0.81, 0.94).

Table 3, Table 4 also show the associations with the control variables. Females (compared to males) reported fewer breakfasts, fewer days of physical activity, more healthful food choices, and fewer unhealthful food choices. Females were also more likely to report dieting to lose weight. Finally, adolescents' age and zBMI both uniquely related to fewer breakfasts, fewer days of physical activity, more unhealthful food choices, and dieting to lose weight.

## 4 Discussion

This study examined the contributions of relative deprivation to lifestyle risk factors for obesity in adolescents. Its focus was not on excess body mass nor obesity per se, but instead on the social determinants of known behavioural contributors to weight problems. With individual differences in gender, age, body mass, school-level absolute deprivation, and subjective social status controlled, we found that relative deprivation within schools related to skipping breakfasts, fewer days of physical activity per week, less healthful food choices, and less dietary restraint. Unhealthful food choices were unrelated to relative deprivation but were related to low absolute affluence and low subjective social status.

These associations with relative deprivation are consistent with previous research on self-rated health in adults (Eibner and Evans, 2005) and psychosomatic symptoms in adolescents (Elgar et al., 2013). The results also support the ecological analysis of income inequality and obesity by Pickett et al. (2005) that posited a psychosocial pathway from increased relative deprivation and intensified social comparisons. In Runciman's (1966) early descriptions of relative deprivation, he wrote that the frustration felt from social comparisons in wealth resulted from the natural tendency for people to compare themselves to those who are better off:

“The magnitude of a relative deprivation is the extent of the difference between the desired situation and that of the person desiring it” (p. 10).

This conceptualisation of deprivation is thus inherently relative and upward-looking in that it involves “a comparison with the imagined situation of some other person or group” (Runciman, 1966, p. 11) called the comparative reference group. Yitzhaki (1979) operationalised the comparisons with a formula that corresponds to the Gini index of inequality. The Yitzhaki index of relative deprivation thus takes into account both individual SEP within a group and the amount of inequality in that group. In applying the Yitzhaki index to adolescents, we assumed that schools are a meaningful comparative reference group and that displays of symbolic capital, like the material possessions and activities that are measured by the HBSC Family Affluence Scale, resonate with adolescents at least as much as income differences. However, there is no consensus in the literature on whether the comparative reference group is best defined by geographic proximity, demographic characteristics, workplaces, or peers (Adjaye-Gbewonyo and Kawachi, 2012). Furthermore, we could not ascertain whether relative deprivation of material assets – a consumption-based measure of affluence – works similarly to relative deprivation of income as described by Yitzhaki and Runciman.

Regarding the other affluence variables in this study, the association found between school-level absolute deprivation and unhealthful food choices is consistent with research on the distribution of convenience stores and fast food restaurants in urban settings, which are more prevalent in lower income neighbourhoods in Canada (Hemphill et al., 2008: Van Hulst et al., 2012) and other countries (Cummins et al., 2005, Hurvitz et al., 2009). The proximity of these establishments to schools has been associated with adolescent health. For example, a study in the United States found that students whose schools were located within a half-mile of a fast food restaurant ate fewer fruits and vegetables, consumed more soft drinks, and were at greater risk of becoming overweight and obese, even after controlling differences found in other student- and school-level characteristics (Currie et al., 2009). Worse still, adolescents who live in more deprived neighbourhoods tend to be less physically active and have poorer aerobic fitness (Charlton et al., 2014). Although not all studies have found such direct associations between the built environment and obesity risk (e.g., Lee, 2012), adolescents whom attended more deprived schools in our study may have been more exposed to obesogenic environments that discouraged healthy food choices and regular physical activity than adolescents attending less deprived schools.

The associations found between subjective social status and obesity risk factors are also consistent with previous studies of adolescent health (Goodman et al., 2003, Quon and McGrath, 2014a). These associations might have been inflated by bidirectional effects on perceived status and self-reported health behaviours (Garbarski, 2010), or because subjective measures of SEP tap into a holistic, internalised status identity that incorporates past and current social circumstances along with future prospects (Pförtner et al., 2015). Other research that examined this unique path to adolescent health has suggested that subjective status could be targeted by interventions to reduce health inequalities between socioeconomic groups, even though subjective and objective SEP seem to relate to health through distinct pathways. Goodman et al. (2007) found that effects of subjective social status on adolescents self-rated health remained strongly significant after differences in parental education were controlled. Similarly, a recent study of adolescents in seven countries found that inequalities in self-rated health and low life satisfaction between levels of subjective social status remained unchanged after differences in family material affluence were controlled (Elgar et al., in press).

Strengths of the study include its large sample and assessment of multiple risk factors for obesity. Limitations include the cross-sectional design and reliance on adolescents' self-reports for estimating body mass indices and dietary behaviours. It would be useful to investigate the long-term health impacts of these SEP variables using longitudinal follow-up given the transactional nature of the SEP-obesity relationship (Gortmaker et al., 1993, Power et al., 2005, Senese et al., 2009). Second, estimates of body mass indices were based on self-reported heights and weights that previous research has found to be biased. The variable correlates highly with BMI based on measured data and was suitable to include here as a control variable, however self-reported BMI cannot accurately differentiate normal weight, overweight, and obese cases using standard cut-point criteria (Elgar et al., 2005). Therefore, we could not explore marginal effects of SEP on risk factors by weight status. Third, the measure of relative deprivation may have included some variation in absolute deprivation within the schools. The high correlation observed between absolute and relative deprivation at the individual level prevented us from including both variables simultaneously in the regression models. Consequently, the associations with relative deprivation may not be entirely psychosocial in nature. This issue has troubled previous investigations of relative deprivation and health (Adjaye-Gbewonyo and Kawachi, 2012) and may have been compounded in this study due to the granularity of the data collected by the HBSC Family Affluence Scale. Fourth, our study relied exclusively on adolescents' self-reports of specific health behaviours, some involving a 7-day recall (physical activity, breakfasts) and others requiring knowledge about specific food types and portion sizes. Although the validity and reliability of these measures have been evaluated previously (e.g., Vereecken and Maes, 2003, Wong et al., 2012), they were still prone to recall errors and biases. Finally, the imputation of missing heights and weights could not account for school-level clustering and this might have compromised the accuracy of some *z*BMI estimates (Reiter et al., 2006).

With these issues in mind, the findings have two main implications for research and practice. First, relative socioeconomic differences between students may present a barrier to obesity prevention, in addition to absolute deprivation in schools and neighbourhoods. The causal chain between relative deprivation and healthy lifestyles is likely to involve stress, which according to a systematic review of socioeconomic differences in obesity is the dominant causal path affecting dietary behaviour in lower SEP groups (Moore and Cunningham, 2012). Efforts to reduce socioeconomic differences in school settings might reduce the intensity of social comparisons, their resultant stress, and negative influences on physical activity and dietary behaviours. Obesity prevention interventions generally focus on access to material resources, like healthy foods, nutritional education, and sports and recreation facilities (Kumanyika and Grier, 2006, Wang et al., 2006). Despite the wealth of evidence on this topic, it is rare to find interventions that acknowledge upstream determinants like economic inequality. It could be that relative deprivation is viewed as an immutable fixture of the economic system or unrelated to health behaviours, but we propose that inequality can be modified and is relevant to reducing health disparities across the life course. Social inequalities in obesity risk in adolescents shape future inequalities in adult obesity-related health problems and should be addressed in school curricula, health promotion interventions, and continued health surveillance efforts.

Second, the findings speak more generally to the multidimensionality of SEP. Three socioeconomic measures captured subjective and objective pathways to health behaviours (i.e., subjective social status and material assets), with the latter further dissected into absolute and relative deprivation indices using the Yitzhaki index. Each path uniquely explained individual differences in obesity risk factors. The dimensions of SEP are not often recognised in health research, and this has generated considerable noise in the literature on poverty, area-level deprivation, and socioeconomic status (Adler and Ostrove, 1999, Goodman et al., 2007). A more nuanced approach to how SEP is conceptualised and measured matters not only to theory development and research advances on health inequalities, but also to practical applications of the evidence.

## Figures and Tables

**Table 1:** Descriptive statistics on key variables (n = 25,980).

Variable	Mean (standard deviation)	Range	95% confidence interval
Age (years)	13.85 (1.52)	9.17, 19.17	13.68, 14.03
Body mass index (*z*BMI)	0.28 (1.14)	−4.91, 4.70	0.25, 0.31
Physical activity (days per week)	4.49 (2.03)	0.00, 7.00	4.40, 4.57
Breakfasts (per week)	5.41 (2.14)	0.00, 7.00	5.32, 5.50
Healthy food choices	4.51 (1.18)	1.00, 7.00	4.47, 4.55
Unhealthy food choices	3.18 (1.00)	1.00, 7.00	3.14, 3.21
Subjective social status	3.68 (1.01)	1.00, 5.00	3.65, 3.71
School-level absolute deprivation	2.90 (0.53)	1.27, 5.89	2.83, 2.97
Relative deprivation	0.92 (0.97)	0.00, 7.50	0.90, 0.93

**Table 2:** Food choices in Canadian adolescents: Percentage and 95% confidence interval.

	Healthful food choices					Unhealthful food choices				
	Fruit	Vegetables	Dark green vegetables	Orange vegetables	Whole grain breads	Sweets, candy, or chocolate	French fries (chips)	Potato chips (crisps)	Soft drinks with sugar	Cake or pastries
Never	1.10 (0.93, 1.30)	1.77 (1.54, 2.04)	10.52 (9.70, 11.41)	4.77 (4.29, 5.31)	4.59 (4.20, 5.01)	1.75 (1.53, 2.01)	7.89 (7.35, 8.46)	7.79 (7.29, 8.31)	10.54 (9.49, 11.69)	7.29 (6.81, 7.79)
Less than once a week	3.87 (3.46, 4.33)	4.07 (3.68, 4.51)	16.08 (15.31, 16.89)	14.40 (13.56, 15.28)	8.45 (7.88, 9.06)	14.23 (13.52, 14.97)	51.18 (49.73, 52.64)	37.40 (36.20, 38.62)	25.66 (24.50, 26.84)	41.23 (39.27, 43.21)
Once a week	6.91 (6.35, 7.51)	7.30 (6.76, 7.87)	18.54 (17.86, 19.25)	20.56 (19.82, 21.33)	9.87 (9.28, 10.49)	18.89 (18.16, 19.73)	22.56 (21.70, 23.44)	24.64 (23.85, 25.46)	18.48 (17.76, 19.22)	23.87 (23.11, 24.65)
2–4 days a week	26.17 (25.17, 27.19)	23.16 (22.32, 24.02)	25.53 (24.62, 26.46)	28.22 (27.23, 29.23)	18.07 (17.33, 18.83)	32.03 (31.24, 32.83)	12.45 (11.69, 13.26)	19.34 (18.46, 20.25)	23.31 (22.40, 24.24)	17.39 (16.30, 18.55)
5–6 days a week	18.32 (17.63, 19.03)	19.97 (19.27, 20.68)	14.56 (13.86, 15.28)	16.33 (15.61, 17.08)	18.98 (18.33, 19.65)	14.91 (14.27, 15.58)	3.57 (3.19, 3.99)	6.32 (5.90, 6.78)	9.92 (9.27, 10.61)	5.61 (4.96, 6.35)
Once a day	19.42 (18.59, 20.29)	23.54 (22.68, 24.42)	8.73 (8.17, 9.33)	9.03 (8.49, 9.61)	20.67 (19.84, 21.53)	9.78 (9.19, 10.40)	1.12 (0.92, 1.38)	2.56 (2.23, 2.93)	5.78 (5.31, 6.29)	2.73 (2.31, 3.23)
More than once a day	24.21 (23.18, 25.28)	20.19 (19.21, 21.20)	6.03 (5.58, 6.52)	6.68 (6.18, 7.22)	19.37 (18.49, 20.27)	8.37 (7.83, 8.93)	1.22 (0.99, 1.50)	1.94 (1.69, 2.24)	6.32 (5.73, 6.97)	1.87 (1.57, 2.23)

Note: These variables were combined into two summary indices, healthful food choices (α = 0.79) and unhealthful food choices (α = 0.76; see Table 1).

**Table 3:** Linear regression analysis of lifestyle risk factors of obesity in Canadian adolescents.

	Breakfasts		Physical activity		Healthful food choices		Unhealthful food choices	
	b (SE)	t	b (SE)	t	b (SE)	t	b (SE)	t
Intercept	5.42 (0.03)		4.50 (0.04)		4.52 (0.02)		3.16 (0.01)	
Gender (female)	−0.47 (0.04)	−11.44**	−0.62 (0.05)	−13.45**	0.16 (0.02)	7.71**	−0.21 (0.02)	−10.32**
Age	−0.23 (0.02)	−13.18**	−0.12 (0.02)	−5.46**	−0.02 (0.02)	−1.77	0.08 (0.01)	10.29**
BMI (z-score)	−0.11 (0.02)	−7.36**	−0.13 (0.02)	−7.22**	−0.04 (0.01)	−3.67**	−0.05 (0.01)	−5.40**
Subjective social class	0.25 (0.02)	12.51**	0.10 (0.02)	4.79**	0.11 (0.01)	9.10**	−0.05 (0.01)	−4.43**
School-level absolute deprivation	−0.23 (0.06)	−4.09**	−0.35 (0.07)	−4.91**	−0.18 (0.04)	−4.65**	0.17 (0.03)	5.63**
Relative deprivation	−0.11 (0.02)	−5.35**	−0.16 (0.02)	−7.25**	−0.10 (0.01)	−8.04**	−0.01(0.01)	−1.13
R^2^		0.07		0.06		0.04		0.04

*p < 0.05. **p < 0.01.

Notes: Shown are slope coefficients (b), standard error of the slope with adjustment for school clustering (SE), and *t*-statistic representing deviation from zero. All six variables were entered to the models simultaneously. BMI = Body mass index. Healthful food choices were fruit, vegetables, dark green vegetables, orange vegetables, and breads with whole grains. Unhealthful food choices were sweets, candy, chocolate, French fries (chips), potato chips (crisps), soft drinks with sugar, and cake or pastries (see Table 2).

**Table 4:** Multinomial logit regression analysis of current dieting: Odds ratio and 95% confidence interval.

Variable	Not dieting, but I should lose some weight (*n* = 4711)	Not dieting because I need to put on weight (*n* = 1979)	Yes, I am dieting to lose weight (*n* = 3197)
Gender (female)	2.62** (2.30, 2.99)	0.41** (0.35, 0.49)	3.49** (3.04, 4.00)
Age	1.12** (1.06, 1.17)	1.12** (1.06, 1.17)	1.18** (1.13, 1.24)
*z*BMI	3.24** (2.99, 3.51)	0.54** (0.50, 0.59)	3.10** (2.85, 3.38)
Subjective social status	0.83** (0.79, 0.88)	0.87** (0.81, 0.94)	0.85** (0.80, 0.90)
School-level absolute deprivation	1.19* (1.03, 1.39)	1.22** (1.07, 1.40)	1.02 (0.90, 1.16)
Relative deprivation	1.00 (0.95, 1.06)	1.05 (0.98, 1.13)	0.94* (0.88, 0.99)

*p < 0.05. **p < 0.01.

Notes: Shown are odds ratios and 95% confidence interval from an unordered, multinomial logit regression model. The most common response to the item on dietary restraint, “No my weight is fine” (*n* = 15,424), was used as the reference category. All six variables were entered to the model simultaneously. Pseudo *R*^*2*^ = 15.1.
